# A case of *Naegleria fowleri* related primary amoebic meningoencephalitis in China diagnosed by next-generation sequencing

**DOI:** 10.1186/s12879-018-3261-z

**Published:** 2018-07-28

**Authors:** Qiang Wang, Jianming Li, Jingkai Ji, Liuqing Yang, Li Chen, Rongrong Zhou, Yang Yang, Haixia Zheng, Jing Yuan, Liqiang Li, Yuhai Bi, George F. Gao, Jinmin Ma, Yingxia Liu

**Affiliations:** 1 Shenzhen Key Laboratory of Pathogen and Immunity, State Key Discipline of Infectious Disease, Shenzhen Third People’s Hospital, 29 Bulan Rd, Shenzhen, 518112 China; 2 CAS Key Laboratory of Pathogenic Microbiology and Immunology, Collaborative Innovation Center for Diagnosis and Treatment of Infectious Disease, Institute of Microbiology, Center for Influenza Research and Early-warning (CASCIRE), Chinese Academy of Sciences, Beijing, 100101 China; 3 BGI-Shenzhen, Shenzhen, 518083 China; 4 Office of Director-General, Chinese Center for Disease Control and Prevention, Beijing, 102206 China; 5 Savaid Medical School, University of Chinese Academy of Sciences, Beijing, 100049 China; 6 China National GeneBank, BGI-Shenzhen, Shenzhen, 518120 China; 7 Laboratory of Genomics and Molecular Biomedicine, Department of Biology, University of Copenhagen, Copenhagen, Denmark

**Keywords:** *Naegleria fowleri*, Primary amoebic meningoencephalitis, Amoeba, Next-generation sequencing

## Abstract

**Background:**

Primary amoebic meningoencephalitis (PAM), caused by *Naegleria fowleri*, is a rare protozoan infectious disease in China. A fatality rate of over 95% had been reported due to extremely rapid disease progression in the USA and other countries. Rapid and precise identification of the causative agent is very important to clinicians for guiding their choices for administering countermeasures in the clinic. In this report, we applied the next-generation sequencing (NGS) method to rapidly show that *N. fowleri* was the causative agent of a fatal case involving a 42-year-old man with severe PAM disease, the first reported in mainland China.

**Case presentation:**

A 42-year old male in a deep coma was admitted to Shenzhen Third People’s Hospital, a special medical care unit with expertise in infectious diseases. Increased intracranial pressure was detected. The cerebrospinal fluid (CSF) sample was found to be red and cloudy with increased leukocyte and protein levels. While bacterial cultures with CSF were negative, *N. fowleri* was determined to be the causative agent with NGS. Amphotericin B (AmB), a drug with anti-amoeba activity, was used immediately, but the treatment came too late and the patient died 2 days after the NGS confirmation.

**Conclusion:**

In this paper, we reported a case of PAM disease for the first time in mainland China. NGS was used for rapid diagnosis and provided guidance for prescribing medications. However, the patient died due to a late admission amid advanced PAM disease. Early detection of *N. fowleri* is necessary in order to select effective drug treatments and control the disease progression. Despite the negative survival outcome, NGS was shown to be a promising method of rapid and precise identification of *N. fowleri*.

**Electronic supplementary material:**

The online version of this article (10.1186/s12879-018-3261-z) contains supplementary material, which is available to authorized users.

## Background

Primary amoebic meningoencephalitis (PAM) is an acute and rapidly fatal disease of the central nervous system, caused by infection with *Naegleria fowleri*, a thermophilic free-living amoeba found in warm freshwater such as lakes, ponds, rivers and hot springs [1]. Contaminated tap water could also be a source of infection [2]. *N. fowleri* usually infects people through the nose cavity when in contact with contaminated water. *N. fowleri* is commonly known as a “brain-eating amoeba” because of its induction of severe encephalitis in the brain upon infection, with a fatality rate of over 95% [3]. There are over 40 species of *Naegleria*, but only *N. fowleri* infections results in PAM [1]. PAM has a rapid, acute disease progression, with an incubation period varying from 2 to 15 days, and death typically occurring 3–7 days after the onset of symptoms [1]. *N. fowleri* has been reported worldwide including America, Australia, Thailand, Hong Kong and Taiwan [4], and about 300 cases in total have been reported in 50 years since the first case was reported in 1965 [3]. Prompt diagnosis is important in order to start treatment as soon as possible. However, prompt diagnosis is not easy for those who had no experience in treatment of *N. fowleri* infection and PAM is often misdiagnosed because no distinctive differences in diagnosis exist to distinguish PAM from bacterial meningoencephalitis [5]. In this study, we report using the next generation sequencing (NGS) method for prompt diagnosis of a *N. fowleri-*related infection, the first case in mainland China.

## Case presentation

On 20th August 2016, a 42-year-old man with a fever (38.4 °C) was admitted to a local hospital in Hangzhou City, China. He lived in Shenzhen City, but was visiting Hangzhou at the time. His disease began with a headache 1 day earlier. Upon admission, blood tests showed a leukocytosis of 10.48 × 10^9^/L (80% neutrophils). On the night of admission, a lumbar puncture showed high levels of white blood cells (WBCs, 1170 cells/ μL, 83% neutrophils), a protein concentration of 3.0 g/L and a glucose concentration of 1.0 mmol/L in the cerebrospinal fluid (CSF) sample. He was treated with 2.0 g of the antimicrobial ceftriaxone and his situation was not improved.

One day later, the patient’s speech became incoherent and he experienced dyspnea. He was transferred to the intensive care unit and endotracheal intubation was applied. He was administered combined therapy comprising of antibiotics (meropenem and linezolid) and an anti-inflammatory drug (dexamethasone). During the night, the patient was found in a coma with dilated bilateral pupils. Computed tomography scans of his brain illustrated hydrocephalus, cerebral edema and foggy brain ventricles. Ventricle puncture for external CSF drainage was performed to reduce intracranial pressure. Four days after admission, microbial culture results of the CSF samples were negative for bacteria and fungi.

**Fig. 1:**
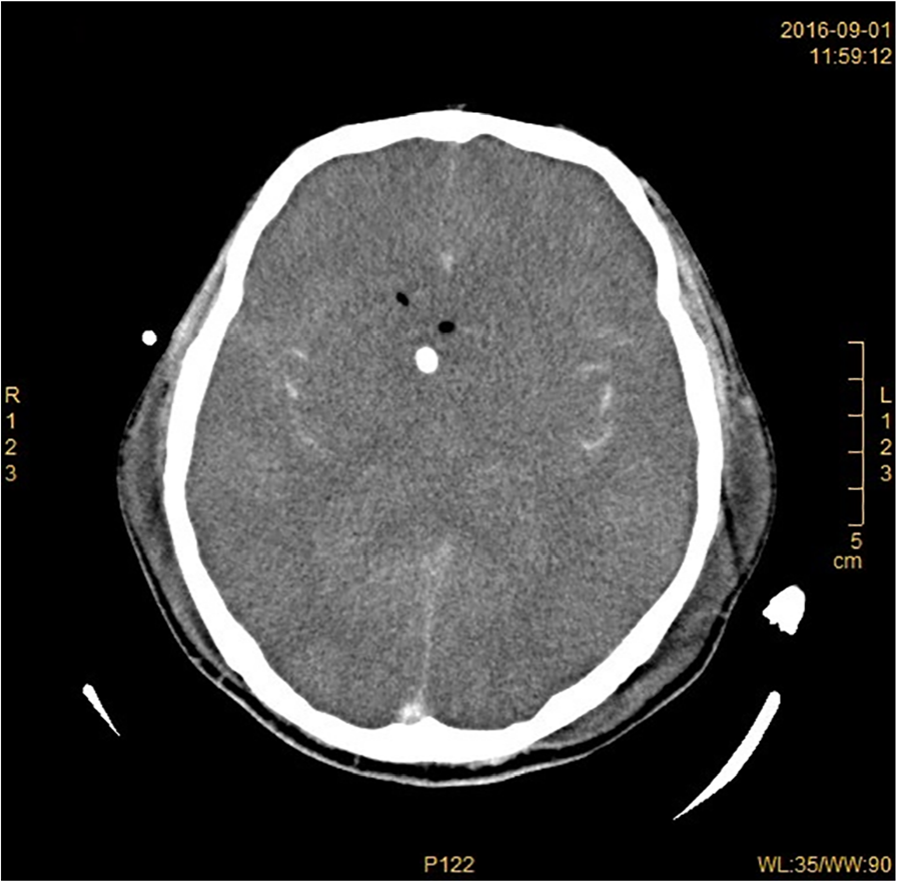
Computed tomography scan of the patient brain with primary amoebic meningoencephalitis

On 31th August, the patient was taken back to Shenzhen at the request of his family, and transferred to Shenzhen Third People’s Hospital, the key institute for handling infectious disease cases. At the time of admission, he was already in a deep coma and unresponsive to simple cues. A blood test showed a leukocytosis of 14.72 × 10^9^ /L (93.3% neutrophils). The CSF appeared light red in color and was mixed with cloudy debris. The WBC count in the CSF had increased substantially to 52,860 cells/ μL (79% lymphocytes), whereas the protein level had increased to 49.96 g/L and the glucose concentration was 0.11 mmol/L. Computed tomography scans showed severe brain swelling. The shape of the cerebral ventricles had become twisted and almost disappeared (Fig. 1).

**Fig. 2:**
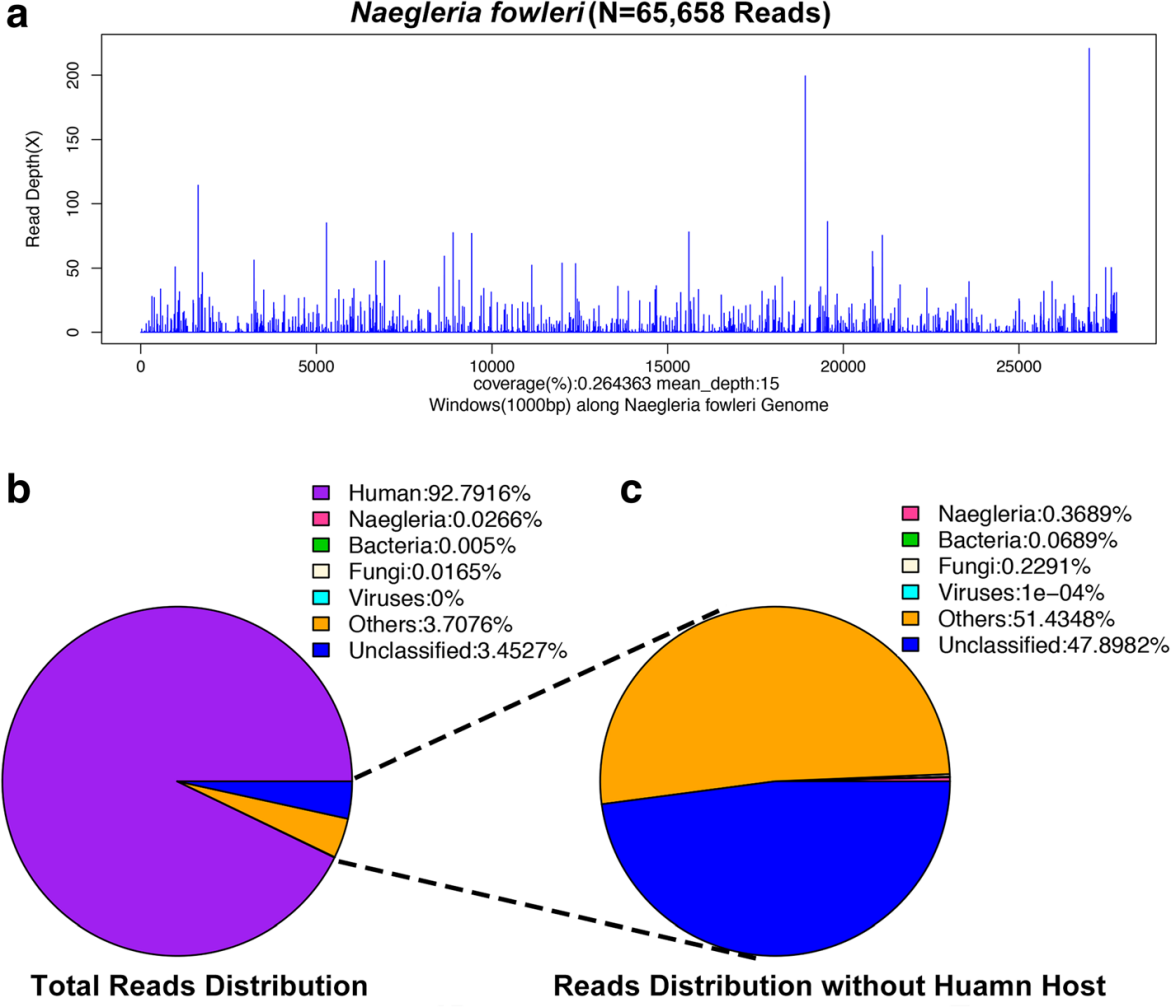
Diagnosis of *N. fowleri* infection using the NGS method. **a**. mapping of *N. fowleri* reads on the genome. **b**. Reads distribution of total DNA in the CSF sample. **c**. Reads distribution of microbes and unknown reads in the absence of human host reads

**Fig. 3:**
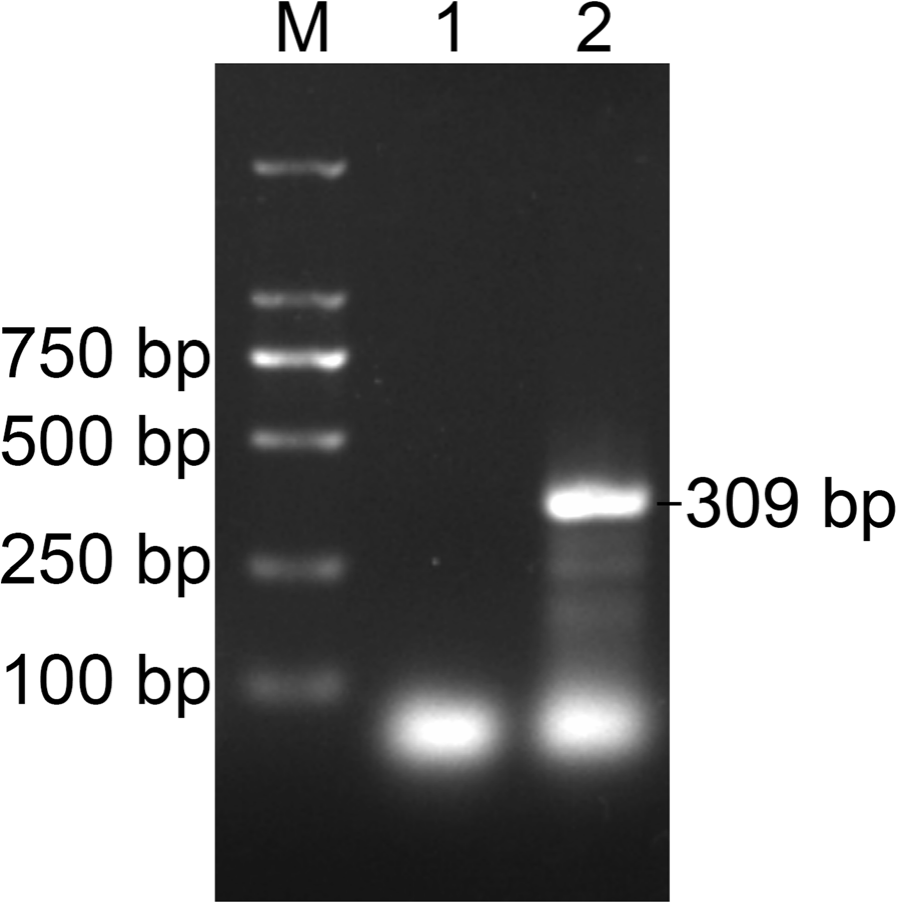
PCR detection of *N. fowleri* from CSF specimen. Lane M: DNA ladder. Lane 1: Negative control. Lane 2: 309-bp PCR product of the 5.8S ribosomal DNA and internal transcribed spacer 2 region of *N. fowleri*

His CSF sample was immediately sent for pathogen detection by NGS at BGI-Shenzhen. Briefly, the CSF sample was centrifuged at 10,000×g for 10 min and the precipitate was used for nucleic acid extraction using the QIAamp DNA Mini Kit (Qiagen), following manufacturer instructions. NGS was performed on the newly developed BGISEQ-500 platform [6]. The sequencing detection identified 65,658 (out of 246,938,950) sequence reads (0.0266%) uniquely corresponding to the *N. fowleri* genome (Fig. 2b) [7], and these reads covered a high percentage of the genome (Fig. 2a). When the reads from the human host were excluded, *N. fowleri* reads were comprised the most of any microbial species, accounting for 0.3689% of total microbial reads and unknown or unclassified reads (Fig. 2c), and more than the total reads of bacterium or fungi (Additional file 1). A 1.6-kb consensus sequence of the 18S ribosomal RNA of *N. fowleri* was assembled (GenBank accession no.KY062165) and found to be 99.99% identical to a reference *N. fowleri* sequence (GenBank accession no. U80059). *N. fowleri* infection was confirmed by PCR (Fig. 3) using the specific primers NFITS-FW (TGAAAACCTTTTTTCCATTTACA) and NFITS-RV (AATAAAAGATTGACCATTTGAAA) covering the 5.8S ribosomal DNA and internal transcribed spacer 2 [4]. Therefore, all these results indicate that the patient was infected with *N. fowleri*. In addition, patient history collected from family members showed that the man was splashed by lake water when he attended the Water-Splashing Festival in Shenzhen approximately 1 weeks before symptoms onset, which may be the source of infection. Anti-amoebic drugs including amphotericin B (AmB) at 50 mg/day and fluconazole 0.4 g/day were immediately administered. Unfortunately, symptoms did not improve and the patient’s family requested to cease medical treatment 2 days later. Life support was withdrawn and the patient was pronounced dead on 3rd September.

## Discussion

We report here a fatal case of PAM caused by *N. fowleri* infection, in which infection likely occurred after the patient had direct contact with lake water 2 weeks before the onset of symptoms. This is the first case of *N. fowleri*-related PAM reported in mainland China. Only two cases were report in Hong Kong and Taiwan, respectively. Although three suspected fatal PAM cases in mainland China had been documented in Chinese journals during 1978, 1990 and 2001, respectively (Table 1), the causative pathogen was never conclusively identified. Increased levels of leukocytes and protein, and a decrease in glucose levels, were consistently observed in all six cases during the disease course.

**Table 1:** Primary amoebic meningoencephalitis cases reported in China and neighboring places

Age	Gender	Location	Year	First CSF after admission					Amoebicidal Treatment	Days from symptom onset to death	Diagnosis
				WBC (cells/μL)	N %	L %	Glucose (mmol/L)	Protein (g/L)			
17	male	Henan^b^	1978	50	5	95	2.95	1.52	NA	10 days	Amoebic trophozoites were observed in the brain tissue after death.
21	male	Beijing^b^	1990	300	70	30	3.4	0. 5	NA	7 days	Amoebic trophozoites were found in the CSF after death.
23	male	Hainan^b^	2001	22,000	85	NA	2.2	2	none	6 days	Amoebic trophozoites were detected in the CSF before death.
38	male	Hong Kong	1993	NA	NA	NA	NA	NA	AmB, rifampicin	survival	Amoeba were observed in an abscess biopsy.
75	male	Taiwan	2011	560	90.5	5	2.2	9.5	AmB	25 days^a^	Amoebic trophozoites were detected in the CSF.
42	male	Shenzhen	2016	1170	83	12	1	3	AmB, fluconazole	15 days	Next-generation sequencing

*CSF* Cerebrospinal fluid, *WBC* White blood cells, *N* Neutrophils, *L* Lymphocytes, *NA* Not available

^a^Days after the start of AmB treatment

^b^Suspected cases documented in Chinese journals without an English citation version

PAM is acute and aggressive, with death typically occurring 3–7 days after onset of symptoms. Early diagnosis and rapid treatment with the appropriate drugs are key factors for effective therapy. In the current case, the man had a suspected incubation period of 5 days and died 15 days after disease onset. Although *N. fowleri* was identified in only 2 days after his admission to Shenzhen Third People’s Hospital, the patient was already in a comatose state, indicating advanced, severe illness. Anti-amoeba medical treatment was initiated 11 days after the onset of disease symptoms; therefore, treatment with the appropriate drugs likely occurred too late to abate the acute phase of PAM.

NGS played a critical role in the accurate diagnosis of *N. fowleri* in the present case. By contrast, the amoeba was not identified in culture. Therefore, NGS constitutes a rapid and accurate method for the identification of pathogens and is particularly helpful in the diagnosis of diseases with unknown causes.

Although no clinically approved specific drugs were available, AmB and miltefosine were reported in successful treatment of *N. fowleri* related PAM. AmB, which is a common drug in clinic and is primarily used as an antifungal medication, has an amoebicidal effect and has been used to treat PAM since 1970. However, the recovery rate with AmB is only 5% (15 recoveries/300 cases worldwide), and this drug can be toxic to humans [3]. Miltefosine, a drug developed against breast cancer and *Leishmania* infections, possesses activity against free-living amoeba species in vitro [8, 9], and is now recommended for the treatment of PAM by the Centers for Disease Control and Prevention in the USA [10], while is not currently available in China. In the United States, 143 PAM cases have been reported, with only four survivors between 1962 and 2016 [11, 12] . Notably, two of the four survivors were related to miltefosine treatment. In 2013 an American girl was diagnosed as PAM about 36 h after symptom onset and was given the recommended miltefosine therapy. She recovered completely without any neurologic impairment [13]. In contrast, an 8 years-old boy was identified with PAM more than 3 days after disease onset. He survived with brain damage although he was also given the recommended miltefosine therapy [14]. Although miltefosine-containing treatment regimen could offer a survival advantage for patients with fatal infections by free-living amoeba, recovery is not assured, since at least three fatal PAM cases were also reported with receiving miltefosine treatment [14, 15]. In addition, miltefosine should be administered in combination with AmB and other drugs, such as rifampin and fluconazole, as soon as possible after diagnosing PAM. In addition to drug treatment, physical procedures like CSF drainage, hyperosmolar therapy, moderate hyperventilation and hypothermia have been employed in the treatment of PAM [3, 16].

Although PAM is a rare disease in China, the rapid disease process and very high fatality rate make it a very dangerous disease. Unfortunately, we have no data about the distribution range of the pathogenic *N. fowleri* in mainland China. More related research should be carried out to analyze the epidemiological risk of *N. fowleri* in China. On the other hand, development of more specific drugs against *N. fowleri* should be put on schedule.

Most previous cases of PAM have been found to have been infected with *N. fowleri* during recreational activities involving freshwater [17]. In the current case, the patient participated in a Water-Splashing Festival several days before the onset of disease; it is therefore likely that contaminated water was the source of infection. This case is a warning that care should be taken when participating in recreational activities involving freshwater due to the possibility of contamination.

## Conclusion

In the current report, a *N. fowleri-*related PAM was rapidly diagnosed using NGS. Although the patient died, the NGS method was shown to be of use to rapidly identify the causative pathogen in the clinic, especially for PAM. The NGS method should be more widely applied in clinical practices, helping physicians on diagnosis and advising on choice of medical countermeasures in the clinic to save lives.

## Additional file

Additional file 1: Microbe reads counts. Other microbe reads including that of bacterium, fungi and virus were listed in the excel table. (XLSX 19 kb)

## Abbreviations

AmB: Amphotericin B
CSF: Cerebrospinal fluid
*N. fowleri*: *Naegleria fowleri*
NGS: Next-generation sequencing
PAM: Primary amoebic meningoencephalitis
